# Contemporary Concepts and Techniques for Scar Minimization in Direct Brow Lift: A Literature Review

**DOI:** 10.3390/jcm15124445

**Published:** 2026-06-09

**Authors:** Ayyad Zartasht Khan, Lars Christian Boberg-Ans, Fredrik Andreas Fineide, Richard Cutler Allen, Elin Bohman, Kim Alexander Tønseth, Hania Nadeem Karamat, Tor Paaske Utheim

**Affiliations:** 1 Department of Ophthalmology, Østfold Hospital Trust, 1535 Moss, Norway; 2 Department of Ophthalmology, Sørlandet Hospital Trust, 4838 Arendal, Norway; 3 Department of Ophthalmology, University Hospital of Southern Denmark, 5230 Vejle, Denmark; 4 Department of Ophthalmology, Innlandet Hospital Trust, 2413 Elverum, Norway; 5 Department of Plastic and Reconstructive Surgery, Oslo University Hospital, 0450 Oslo, Norway; 6 Department of Medical Biochemistry, Oslo University Hospital, 0450 Oslo, Norway; 7 The Norwegian Dry Eye Clinic, Ole Vigs Gate 32 E, 0366 Oslo, Norway; 8 Department of Computer Science, Oslo Metropolitan University, 0176 Oslo, Norway; 9 Department of Ophthalmology, Baylor College of Medicine, Houston, TX 77030, USA; 10 Oculoplastic and Orbital Services, St. Erik Eye Hospital, 171 64 Stockholm, Sweden; 11 Department of Clinical Neuroscience, Division of Eye and Vision, Karolinska Institutet, 171 65 Solna, Sweden; 12 Institute for Clinical Medicine, Faculty of Medicine, University of Oslo, 0371 Oslo, Norway; 13 Department of Trauma and Orthopaedic Surgery, Ysbyty Gwynedd Hospital, Bangor LL57 2PW, UK; 14 Department of Ophthalmology, Oslo University Hospital, 0450 Oslo, Norway; 15 Department of Ophthalmology, Stavanger University Hospital, 0101 Oslo, Norway; 16 Department of Ophthalmology, Vestre Viken Hospital Trust, 3004 Drammen, Norway; 17 Department of Ophthalmology, Vestfold Hospital Tønsberg, 3103 Tønsberg, Norway; 18 Department of Life Sciences and Health, Oslo Metropolitan University, 0176 Oslo, Norway; 19 Department of Clinical Medicine, Faculty of Medicine, University of Bergen, 5007 Bergen, Norway; 20 Department of Quality and Health Technology, The Faculty of Health Sciences, University of Stavanger, 4021 Stavanger, Norway; 21 Department of Oral Biology, Faculty of Dentistry, University of Oslo, 0371 Oslo, Norway; 22 National Centre for Optics, Vision and Eye Care, Department of Optometry, Radiography and Lighting Design, Faculty of Health Sciences, University of South-Eastern Norway, 3679 Kongsberg, Norway; 23 Department of Health and Nursing Science, The Faculty of Health and Sport Sciences, University of Agder, 4630 Grimstad, Norway; 24 Department of Ophthalmology, Faculty of Life Course Sciences and Medicine, King’s College London, London WC2R 2LS, UK

**Keywords:** direct brow lift, oculofacial plastic surgery, scar minimization, scar visibility, brow ptosis, eyebrow lift

## Abstract

**Background**: Despite the rise of endoscopic approaches, the direct brow lift remains one of the most effective procedures for correcting brow ptosis for both functional and cosmetic indications. It continues to offer superior control when correcting brow shape, height, and asymmetry. However, visible scarring remains a concern. This systematic review was conducted to synthesize recent evidence on strategies that minimize visible scarring in direct brow lift surgery. **Methods**: A systematic literature search was performed to retrieve English-language publications from the past decade, discussing scar-minimization strategies in direct brow lift. A total of 124 records were identified through database searches in Ovid MEDLINE and Embase. Records were screened manually according to predetermined criteria, and those not in English, not addressing scarring, or not focused on direct brow lift were excluded. After this process, ten publications were included in the final qualitative synthesis. **Results**: The qualitative synthesis of all included publications (together comprising data on approximately 900 patients) revealed several strategies for scar minimization. (1) Incision beveling: A shallow cranially directed bevel between 20° and 45° preserves brow hair follicles and allows hair regrowth through the scar, providing natural camouflage. (2) Undermining: Gentle subcutaneous undermining in a limited 1–2 cm field, while preserving subcutaneous fat, allows tension-free advancement and maintains brow volume. (3) Periosteal suspension: Anchoring the mobilized brow flap to the frontal periosteum redistributes tension away from the dermal closure, maintaining elevation and improving scar quality. (4) Layered closure: Two- or three-layered wound closure with deep dermal anchoring and fine everting skin sutures minimizes dermal traction and scar widening. (5) Adjunctive measures: Evidence for topical silicone gel was inconclusive, whereas postoperative laser therapy and perioperative neuromodulator use demonstrated improved scar appearance. Across studies, outcomes were consistent, with high patient satisfaction, inconspicuous scars in over 85% of cases, and low complication or revision rates. **Conclusions**: Direct brow lift has historically been criticized for conspicuous scarring, contributing to the popularity of endoscopic techniques. Nevertheless, the traditional direct brow lift remains a fundamental skill in the oculofacial plastic surgeon’s armamentarium, offering unmatched accuracy in brow repositioning, reliability, and symmetry. Contemporary evidence demonstrates refinements that can markedly minimize scar visibility. This systematic review and qualitative synthesis allow us to continue to refine and improve our techniques to minimize scarring in direct brow lift to the benefit of our patients.

## 1. Introduction

The position and contour of the eyebrows play an important role in the visual field [1], social settings [2], and facial esthetics [3]. Brow ptosis, whether age-related, secondary to trauma, or due to facial nerve dysfunction, can have negative implications for quality of life [4]. Over the past century, numerous surgical techniques have been developed for the correction of brow position and contour [5]. These include endoscopic, coronal, pretrichial, and transblepharoplasty approaches. Nevertheless, the direct brow lift remains the most powerful method for brow lift, offering accurate control of both brow height, shape, and contour [6].

Despite its precision and effectiveness, the direct brow lift continues to be limited by the potential for visible postoperative scarring. Because the incision is placed in an exposed area immediately above the brow, even a fine linear scar can be noticeable, particularly in patients with thin or sparse brow hair. This makes patient selection and preoperative counseling particularly important. Thick sebaceous brow skin, often encountered in male patients, may increase the risk of a more visible scar or contour irregularity, whereas thin or thinning brow hair may reduce the ability of the brow to camouflage the incision. These factors should therefore be considered when selecting patients, planning incision placement, and counseling patients about scar visibility. Variations in individual healing characteristics and skin type further influence scar visibility. These esthetic concerns have significant medicolegal implications: brow lift surgery ranks among the most commonly litigated procedures in oculoplastic practice, with excessive or disfiguring scarring cited as the most frequent cause of malpractice claims [7]. Such outcomes can lead to patient dissatisfaction, revision requests, and legal disputes, highlighting the importance of surgical technique and thorough preoperative counseling. Although alternative approaches such as endoscopic and pretrichial brow lifts have gained popularity for their concealed incisions, the direct brow lift remains indispensable in cases requiring precise control of brow shape, symmetry, and elevation. The challenge, therefore, lies not necessarily in the indication for surgery but in refining the technique to achieve consistent, durable, and esthetically subtle results while minimizing scar conspicuity.

This systematic literature review consolidates contemporary evidence on surgical and adjunctive strategies intended to minimize scarring in direct brow lift surgery. The aim is to identify reproducible principles that may help surgeons achieve less conspicuous scars while preserving functional and esthetic control of brow position.

## 2. Methods

**Figure 1:**
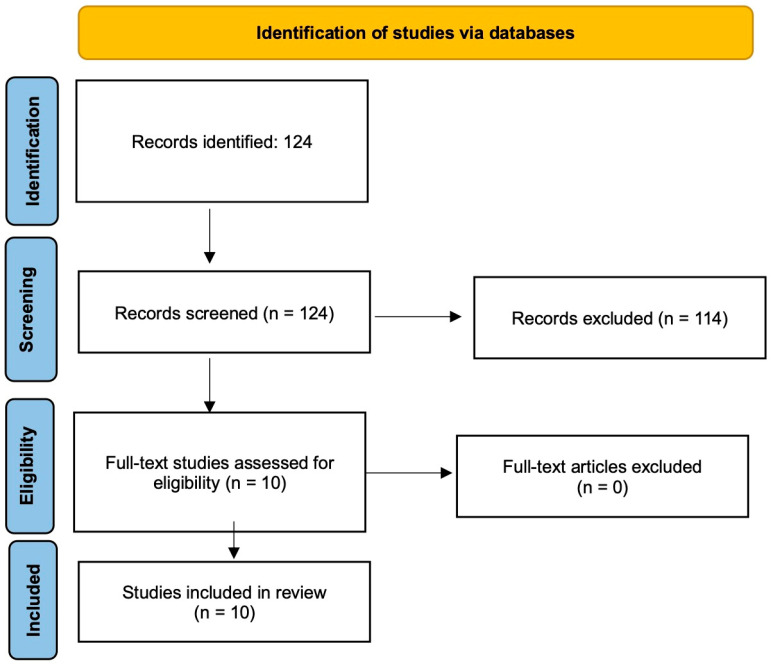
A Preferred Reporting Items for Systematic Reviews and Meta-Analyses (PRISMA) flow diagram of study identification, inclusion, and exclusion.

A systematic literature search was conducted in Ovid MEDLINE and Embase on 13 March 2025 by a medical librarian experienced in systematic searching. The search combined terms for brow lifting and direct brow lift (including brow lift, eyebrow lift, brow ptosis, and brow incision) with scar-related terms (including scar, cicatrix, and keloid). The search was limited to English-language publications from the preceding 10 years. The complete search strategy is provided as Supplementary Materials (Supplementary Materials Table S1). Studies were eligible if they focused on the direct brow lift and reported scar outcomes or technical measures relevant to scar visibility, including incision design, dissection plane, fixation or tension reduction, wound closure, or adjunctive scar management. Prospective and retrospective cohorts, randomized trials, surveys, and technique papers were considered. Studies were excluded if they did not address scarring, focused primarily on endoscopic, temporal, coronal, or other nondirect approaches without separate direct brow lift data, or were not published in English. The initial search retrieved 124 records. Two reviewers independently screened all titles and abstracts; this was followed by full-text assessment of potentially eligible studies. Records were excluded if they (a) did not primarily address scar minimization or scar outcomes following brow lift procedures (*n* = 87), (b) focused on endoscopic, temporal, or coronal approaches as opposed to the direct brow lift technique (*n* = 26), or (c) were not published in English (*n* = 3). Reference lists of all included papers were manually searched to identify additional eligible studies not captured in the electronic search. Subsequently, ten publications remained eligible and were included in the final qualitative synthesis (Figure 1). These comprised a survey of surgeons, prospective cohort studies, retrospective cohort studies in patients with facial palsy, a randomized controlled trial comparing silicone gel with placebo, technique and position papers, and additional prospective cohorts and retrospective analyses addressing scar-minimization strategies specific to direct brow lift surgery. During the preparation of this manuscript, the authors used an AI-based language model (ChatGPT, version 5.0 Pro) to assist with English-language editing and grammatical refinement. The authors have reviewed and edited the output and take full responsibility for the content of this publication.

## 3. Results

**Figure 2:**
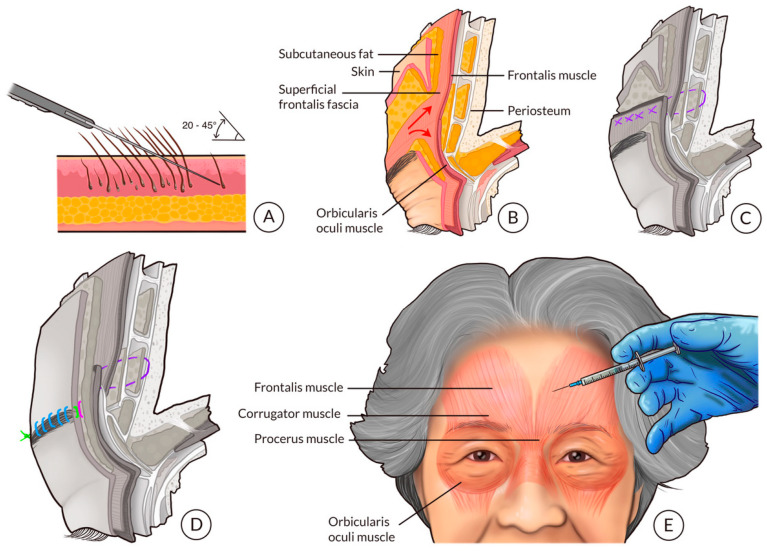
Strategies repeatedly identified across the reviewed literature for achieving inconspicuous scarring. A shallow cranially directed bevel between 20° and 45° preserves hair follicles and allows regrowth through the incision line, providing natural camouflage (**A**). Gentle subcutaneous undermining in a limited 1–2 cm field, while preserving subcutaneous fat, facilitates tension-free advancement and maintains brow volume (**B**). Periosteal suspension redistributes tension away from the dermal closure, maintaining brow elevation and preventing scar widening (**C**), followed by layered closure with fine everting sutures for optimal dermal alignment (**D**). Adjunctive perioperative botulinum toxin injections reduce dynamic muscle tension during healing, supporting improved scar quality (**E**). Parts of this figure were adapted and modified with inspiration from Fakih-Gomez et al., Feinendegen et al., and Abraham et al. Illustration: Kristin Skårdal.

Ten publications met the inclusion criteria: prospective and retrospective cohorts or analyses, randomized trials, technique-oriented reports, and one survey of surgeons. Patient-based studies ranged from single-case technical reports to cohorts of 496 patients; the surgeon survey included 276 respondents. Across studies, the most frequent scar-minimizing themes were incision beveling parallel to brow follicles, limited undermining with preservation of subcutaneous volume, deeper fixation to reduce closure tension, layered closure with skin-edge eversion, and selected adjuncts such as neuromodulators or laser therapy (Figure 2). Quantitative scar-related reporting was inconsistent. Shu et al. reported “inconspicuous scars” in 428 of 432 followed patients [8]; Pascali et al. reported “statistically significant” brow elevation in 98% of patients with visible scarring in 2 of 50 cases [9]; Butler et al. reported a mean Manchester Scar Scale score of 8.6/28, no postoperative forehead paraesthesia or brow alopecia, and “excellent” or “good” brow height in 85% of brows [10]; Fakih-Gomez et al. reported symmetrical brow positioning in 97.8% of patients with generally “inconspicuous” scarring [11]; and Feinendegen et al. reported “very good” or “good” outcomes in 16 of 18 patients. Cadet et al. found no significant advantage of topical silicone gel over placebo [12], whereas Tenzel et al. found patient-rated improvement after serial non-ablative Nd:YAG laser sessions [13]. Patient selection was variably reported across the included studies. Some studies defined cohorts according to indication or population subgroup, including facial palsy-related brow ptosis, Asian women with lateral brow ptosis and upper eyelid hooding, men undergoing direct brow lift with upper blepharoplasty, and patients enrolled for postoperative scar treatment. However, few studies explicitly selected or stratified patients according to scar-relevant anatomical factors such as brow skin thickness, brow hair density, thinning lateral brow hair, sebaceous skin quality, or predisposition to hypertrophic scarring. Smoking was reported as a relevant negative factor in one study, but systematic reporting of patient-selection criteria related to scar camouflage was generally limited. Study-level details, including reported incision extent and associated refinements, are summarized in Table 1.

In summary, we found the following concepts to be repeated in the majority of our included full texts: (1) incision beveling, (2) gentle undermining, (3) periosteal suspension, (4) layered suturing with skin-edge eversion during final skin sutures, and (5) the use of perioperative neuromodulators (Figure 2).

## 4. Discussion

By describing the excision of small, symmetrical ellipses of skin immediately above each brow to achieve elevation, Dr. Raymond Passot is credited with popularizing the direct brow lift in the 1930s [18]. Since then, numerous modifications and alternative approaches, including endoscopic, coronal, pretrichial, and transblepharoplasty techniques, have been developed to refine outcomes and reduce morbidity [5]. Despite these advances, the direct brow lift remains useful because it offers predictable control over brow height, shape, and symmetry [10]. However, the visible incision makes scar management central to patient selection, counseling, and operative planning [12].

Preoperative marking deserves specific attention because the shape and extent of excision influence both brow contour and scar concealment. The reviewed techniques vary from classical elliptical or fusiform suprabrow excisions, often with the maximal vertical height positioned near the lateral third of the brow arch, to more laterally weighted markings designed primarily to elevate the lateral brow tail. The medial and lateral limits of the excision are also important. Medial brow skin is often thicker and may be less forgiving if over-resected, whereas lateral brow hair may be thinner and provide less reliable camouflage. Therefore, the marking should be individualized according to the vector of brow ptosis, desired brow shape, brow hair density, skin thickness, sex-associated brow esthetics, and the need for either global or predominantly lateral elevation.

Incision design is one of the most consistently reported determinants of scar concealment. The reviewed studies describe either full-length supraciliary excisions when global elevation or asymmetry correction is required, or segmental and lateral designs when the deformity is mainly temporal or lateral. Regardless of extent, the incision is generally placed at or within the superior brow margin and beveled parallel to hair follicles. Feinendegen et al. used a 20° cranially beveled incision just below the superior brow margin to include the upper rows of brow hair [17]. In a subsequent publication, the authors further elaborated on the “flat incision technique”, showing that a shallow, cranially directed bevel increases the dermal surface area available for wound healing and enhances scar quality [15]. Similarly, Pelle-Ceravolo and Angelini employed a 30° bevel and limited undermining, confirming that shallow incision angles minimize hair bulb transection and improve scar concealment [16]. Together, these studies confirm that shallow cranially directed beveling between 20° and 45° remains an important strategy for minimizing scar visibility in direct brow lift surgery. Incision extent and contour should also reflect patient anatomy and sex-associated esthetic goals. In men, a lower and flatter brow contour is often desirable, and denser brow hair may permit a longer supraciliary incision to be camouflaged, as illustrated by Pascali et al. [9]. In women, especially when the goal is restoration of a lateral peak rather than global elevation, segmental or lateral designs may reduce the risk of over-elevating the medial brow. Shu et al. further showed that in Asian women, who may have higher baseline brows, wider upper eyelids, and more upper eyelid fullness, a combined supra-brow and infra-brow design can address lateral hooding while avoiding excessive brow elevation [8]. These observations support individualized incision planning rather than a uniform lateral or full-length approach.

Gentle subcutaneous undermining was another consistently emphasized element across the reviewed studies. The rationale behind undermining is to free the brow flap from the underlying orbicularis and frontalis fascia to reduce the risk of brow ptosis relapse, to allow tension-free advancement, and to prevent traction and tension on the final wound (which would cause excessive scarring). Most authors describe subdermal undermining in a narrow field, typically 1–2 cm cranially and caudally from the incision, in a plane superficial to the frontalis and orbicularis fascia [9,15,16,17]. Feinendegen et al. performed subdermal undermining in both directions within this limited range to achieve smooth contouring [15,17]. Similarly, Pascali et al. restricted dissection to approximately 7–10 mm below the brow and 2–3 mm above it [9]. Pelle-Ceravolo and Angelini supported this conservative approach, performing limited subdermal undermining only to relieve tension on closure [16]. Interestingly, Fakih-Gomez et al. describe a more excessive deep-plane undermining performed not only in the subcutaneous plane, but also beneath the subfrontalis fascia (via transection of orbicularis oculi muscle) until it reaches the retro-orbicularis fat and downward until reaching the orbital retaining ligament region [11]. After all this release, the brow is suspended to the frontal periosteum by Vicryl sutures passed through the orbicularis muscle and the deep frontalis fascia [11]. Throughout the dissection, the subcutaneous fat is preserved to maintain brow volume and avoid contour deformities, a principle highlighted across several studies. Regardless of the extent of undermining, all studies emphasized the importance of not damaging the brow hair follicles during dissection. Similarly, hemostasis was also stressed.

Periosteal suspension aims to anchor the mobilized brow flap to the underlying frontal periosteum, thereby maintaining brow elevation and preventing scar widening caused by tension. It also transfers tension from the dermal closure to a deeper, more stable fixation point. When performed appropriately, periosteal suspension should be achieved without skin puckering. Butler and colleagues demonstrated that small transcutaneous punctures for periosteal fixation could provide stable support without added dissection or contour irregularity, yielding excellent scar and sensory outcomes in patients with facial palsy [10]. Fakih-Gomez et al. employed, following extensive undermining, a superior anchoring suture to the frontal periosteum by traversing through the frontalis muscle at the maximum point of elevation [11]. This was in addition to 3–5 suspending sutures passed in the same manner, but more inferiorly. Feinendegen et al. [15,17] and Pascali et al. [9] also incorporated periosteal sutures to distribute tension evenly across the flap while limiting closure stress. Periosteal suspension not only enhances longevity of elevation but also contributes to improved scar esthetics by reducing wound-edge traction. Across all reviewed studies, periosteal suspension was consistently identified as an effective strategy to ensure durable brow positioning and superior scar quality. A recent case series, published after the prespecified search date, extends this concept to retro-orbicularis oculi fat (ROOF) mobilization and superior fixation [19]. Murdock et al. reported 9 male patients undergoing bilateral direct brow lift with ROOF repositioning; mean brow-reflex distance improved from 5.17 to 11.76 mm, temporal hooding improved in all patients, and no revisions, frontalis paresis, or neural compromise were reported [19]. Although this study primarily addresses functional and contour outcomes rather than scar scoring, it highlights the intraoperative value of deeper soft-tissue support and volume repositioning, particularly in mature male patients.

Layered closure with skin-edge eversion is another recurring scar-minimizing principle. The purpose of a layered wound closure is to distribute tension evenly across the wound, minimize dermal traction, and achieve precise skin-edge apposition for fine, inconspicuous scars. Most authors describe a two- or three-layer closure beginning with deep dermal sutures followed by fine subcuticular or cutaneous sutures for accurate epidermal alignment [9,15,16,17]. Feinendegen et al. used deep 5-0 Maxon sutures grasping periosteum, combined with a continuous 7-0 Prolene skin suture [15,17]. Pelle-Ceravolo and Angelini emphasized skin-edge eversion and the use of fine nylon sutures to prevent track marks and scar depression [16]. Interestingly, Butler et al. describe counter-bevelling of the upper and lower incision lines so that, upon closure of the deep dermis, the wound edges are encouraged to pout [10]. However, this is in contrast to the beveling technique described above to preserve hair follicles. Overall, the reviewed evidence supports multilayered closure with careful tension distribution and skin-edge eversion in achieving imperceptible scars. This is also in line with established surgical practice.

Beyond refinements in surgical technique, adjunctive strategies to improve scar remodeling have also been explored. Cadet et al. conducted a randomized controlled trial comparing silicone gel to placebo following direct brow lift and found no statistically significant difference in scar appearance between the two groups, suggesting that topical silicone gel alone may have limited benefit [12]. Tenzel et al. demonstrated that postoperative use of non-ablative 1064 nm Nd:YAG laser enhanced scar appearance after direct brow lift [13]. The perioperative use of neuromodulators represents another increasingly recognized adjunct in optimizing scar outcomes [20]. The rationale lies in the ability of botulinum toxin to induce temporary chemodenervation of the frontalis, corrugator, procerus, and orbicularis oculi muscles, thereby reducing dynamic tension across the healing wound. Constant micromovement in the early postoperative phase has long been associated with widened or hypertrophic scars, and selective neuromodulation mitigates this by maintaining relative immobility during the critical period of collagen deposition and remodeling. Abraham et al. surveyed 276 surgeons and found that approximately 25% routinely administered neuromodulators two weeks before surgery and 45% continued treatment postoperatively, typically every three months, to sustain tension reduction during healing [14]. Fakih-Gomez et al. also incorporated preoperative botulinum toxin injections to the depressor supercilii and orbicularis oculi muscles, complementing mechanical tension release achieved through deep-plane dissection [11]. Similarly, Pelle-Ceravolo and Angelini injected 5 units of botulinum toxin into the frontalis muscle above the lateral two-thirds of each brow two to three days before surgery [16]. These findings mirror a broader trend in esthetic and reconstructive surgery, where neuromodulators are increasingly employed perioperatively to optimize scar outcomes following blepharoplasty, facelift, and resurfacing procedures [21]. Collectively, current evidence supports the judicious use of perioperative neuromodulators as a clinically effective adjunct to traditional scar-minimizing techniques in direct brow lift surgery.

An important limitation across the reviewed studies is the variability in how outcomes, particularly scar outcomes, are assessed. In many cases, results are described using subjective terms such as “minimal”, “inconspicuous”, or “imperceptible”, without clear definitions or standardized criteria. Objective characteristics such as scar width, depth, length, color, and location are only inconsistently reported, making it difficult to compare findings across studies. Similarly, validated scar assessment tools, such as the Vancouver Scar Scale or the Patient and Observer Scar Assessment Scale, are rarely used. Patient-reported outcomes are commonly included, but these are often limited to general satisfaction or cosmetic acceptability and are typically measured using non-standardized questionnaires. This lack of consistency introduces potential bias and limits the strength of conclusions that can be drawn from the existing literature.

A closer look at the included studies further illustrates this variability. Some authors incorporated objective measurements. For example, Pascali et al. used standardized photographic analysis to quantify brow position and combined this with both clinician and patient-based assessments [9]. Shu et al. similarly reported measurable periocular parameters, such as palpebral fissure height and eyebrow-to-eyelid distances, alongside complication rates and patient satisfaction [8]. In contrast, other studies relied more heavily on descriptive evaluations based on clinical photographs and overall impressions of scar visibility. A few studies attempted more structured assessments; for instance, Tenzel et al. used numerical rating scales and blinded photographic comparisons, but these still largely reflected perceived appearance rather than detailed scar morphology [13]. Across the literature, patient-reported outcomes were almost always included, yet the way they were collected and reported varied widely. Taken together, while many studies attempt to capture both objective and subjective outcomes, the lack of standardized, multidimensional assessment frameworks remains a key limitation.

In conclusion, contemporary refinements in direct brow lift surgery reflect a clear evolution toward techniques that balance functional precision with esthetic subtlety. The available evidence suggests that optimal scar outcomes are achieved through a combination of careful incision design, controlled tissue handling, tension redistribution, and thoughtful use of adjunctive therapies. At the same time, future research would benefit from more consistent and comprehensive outcome assessment, integrating both objective scar characteristics and validated patient-reported measures. Such standardization would allow more meaningful comparisons across studies and further refine best practices in this procedure.

## Figures and Tables

**Table 1:** Summary of included publications evaluating scar-minimization strategies in direct brow lift surgery. Incision extent is described as reported by the included publications; when lateral versus full-length extent was not explicitly quantified, this is indicated as individualized or NR (not reported). Abbreviations: OOM, orbicularis oculi muscle; Nd: YAG, neodymium-doped yttrium aluminum garnet; RCT, randomized controlled trial.

No.	Study	Design/*n*	Incision Extent/Design	Key Refinements	Reported Outcome
1	Abraham et al. [14]	Survey/276 surgeons	N/A	Perioperative neuromodulator practice	25% preoperative; 45% postoperative; repeats commonly every 3 months
2	Fakih-Gomez et al. [11]	Prospective cohort/45	Direct suprabrow incision; individualized extent	Deep-plane release; orbicularis suspension; periosteal fixation; preoperative neuromodulator	Minimal scarring; 97.8% symmetrical brow position; transient sensory symptoms resolved
3	Butler et al. [10]	Retrospective cohort/23 pts; 24 brows	W-plasty suprabrow incision; extent tailored to ptosis	Counter-beveled edges; subcutaneous-only excision; periosteal browpexy via small frontalis punctures	Manchester Scar Scale 8.6/28; no paraesthesia/alopecia; excellent/good brow height in 85%; 1 temporal revision
4	Cadet et al. [12]	RCT split-scar/12 pts; 24 scars	Bilateral direct brow lift scars; extent NR	Silicone gel vs. placebo for 2 months	No significant scar benefit from silicone gel
5	Feinendegen et al. [15]	Technique report/1	20° cranially beveled incision within superior brow margin; extent dictated by defect	Follicle-preserving lower incision; 1–2 cm release; periosteal suspension; layered closure	Increased dermal contact area; favorable scar quality reported
6	Pelle-Ceravolo and Angelini [16]	Prospective cohort/212	Transcutaneous brow shaping; segmental extent individualized	30° bevel; deep dermis/fat preservation; limited undermining; preop botulinum toxin; everted layered closure	Low scar visibility and high satisfaction; no major complications
7	Feinendegen et al. [17]	Prospective cohort/18	20° beveled brow incision; extent tailored to reduction/reshaping	Follicle preservation; 1–2 cm undermining; periosteal suspension; running skin suture	Very good/good in 16/18; poor results in smokers; no motor/sensory disorders
8	Tenzel et al. [13]	RCT split-face laser/9	Postoperative direct browplasty scars; extent NR	Six unilateral 1064-nm Nd:YAG laser sessions	Patient-rated improvement significant; masked observers more subtle; transient mild/moderate effects; no hair loss
9	Shu et al. [8]	Prospective cohort/496; 432 follow-up	Combined supra-/infra-brow swallow-tail excision from medial brow to tail	Follicle-parallel incisions; OOM excision/suspension; periosteal fixation; limited undermining; layered closure	Inconspicuous scars in 428/432 (99.1%); 94.7% satisfaction; no facial nerve injury; 8 transient numbness cases
10	Pascali et al. [9]	Retrospective analysis/50	Full-length lift of medial, central, and lateral brow	Follicle-parallel incision; 7–10 mm undermining; no orbicularis incision; everted layered closure	Significant elevation in 98%; visible scar in 2/50; 12–24 month follow-up

## Data Availability

No new data were created or analyzed in this study.

## Supplementary Materials

The following supporting information can be downloaded at: https://www.mdpi.com/article/10.3390/jcm15124445/s1, Table S1: Search strategy used for the systematic literature review on scar-minimization techniques in direct brow lift surgery. In both databases, a forward slash (/) following a term indicates a controlled subject heading, while the term “.ti,ab,kf” restricts the search to title, abstract, or author keywords. The asterisk (*) denotes truncation, allowing retrieval of all word variants (e.g., scar retrieves scar, scars, scarred, and scarring). The command exp explodes a subject heading to include all narrower terms within the hierarchy. The adjacency operator (“adj” or “adj5”) retrieves records in which the specified words appear next to each other or within five words of one another, in any order.
