# Supplementary material for: Contemporary Concepts and Techniques for Scar Minimization in Direct Brow Lift: A Literature Review
Source: J Clin Med. 2026 Jun 9;15(12):4445. doi: 10.3390/jcm15124445 (PMC13301638; doi:10.3390/jcm15124445)
Supplement: Supplementary file 1 [file jcm-15-04445-s001.zip › jcm-4312725-supplementary.pdf]

Supplementary Table S1.

	**Ovid MEDLINE search**	**Embase search**
1	(brow lift* or browlift or eyebrow lift* or eyebrowlift*).ti,ab,kf.	(brow lift* or browlift or eyebrow lift* or eyebrowlift*).ti,ab,kf.
2	((brow* or eyebrow*) adj5 (lift* or elevat*)).ti,ab,kf.	((brow* or eyebrow*) adj5 (lift* or elevat*)).ti,ab,kf.
3	((brow* or eyebrow*) adj ptosis).ti,ab,kf.	((brow* or eyebrow*) adj ptosis).ti,ab,kf.
4	((brow* or eyebrow*) adj5 incision*).ti,ab,kf.	((brow* or eyebrow*) adj5 incision*).ti,ab,kf.
5	or/1-4	or/1-4
6	exp Cicatrix/	exp Scar/
7	(cicatri* or scar* or keloid*).ti,ab,kf.	(cicatri* or scar* or keloid*).ti,ab,kf.
8	or/6-7	or/6-7
9	5 and 8	5 and 8
10	(direct brow or direct browlift* or direct eyebrow*).ti,ab,kf.	(direct brow or direct browlift* or direct eyebrow*).ti,ab,kf.
11	9 or 10	9 or 10

Supplementary Table S1. Search strategy used for the systematic literature review on scar-minimization techniques in direct brow lift surgery. In both databases, a forward slash (/) following a term indicates a controlled subject heading, while the term ".ti,ab,kf" restricts the search to title, abstract, or author keywords. The asterisk (*) denotes truncation, allowing retrieval of all word variants (e.g., scar retrieves scar, scars, scarred, and scarring). The command exp explodes a subject heading to include all narrower terms within the hierarchy. The adjacency operator ("adj" or "adj5") retrieves records in which the specified words appear next to each other or within five words of one another, in any order.
